# Single versus multimodality training basic laparoscopic skills

**DOI:** 10.1007/s00464-012-2184-9

**Published:** 2012-02-21

**Authors:** Willem M. Brinkman, Sanne Y. Havermans, Sonja N. Buzink, Sanne M. B. I. Botden, Jack J. Jakimowicz, Benedictus C. Schoot

**Affiliations:** 1 Department of Urology, Catharina Hospital Eindhoven, Michelangelolaan 2, 5623 EJ Eindhoven, The Netherlands; 2 Department of Obstetrics and Gynecology, Catharina Hospital Eindhoven, 5623 EJ Eindhoven, The Netherlands; 3 Faculty of Industrial Design Engineering, Delft University of Technology, 2628 CE Delft, The Netherlands; 4 Department of Surgery, Maastricht University Medical Centre, 6202 AZ Maastricht, The Netherlands; 5 Department of Surgery, Catharina Hospital Eindhoven, 5623 EJ Eindhoven, The Netherlands

**Keywords:** Laparoscopic training, Modality, Simulators, Virtual reality, Practice variability

## Abstract

**Introduction:**

Even though literature provides compelling evidence of the value of simulators for training of basic laparoscopic skills, the best way to incorporate them into a surgical curriculum is unclear. This study compares the training outcome of single modality training with multimodality training of basic laparoscopic skills.

**Methods:**

Thirty-six medical students without laparoscopic experience performed six training sessions of 45 min each, one per day, in which four different basic tasks were trained. Participants in the single-modality group (S) (*n* = 18) practiced solely on a virtual reality (VR) simulator. Participants in the multimodality group (M) (*n* = 18) practiced on the same VR simulator (2x), a box trainer (2x), and an augmented reality simulator (2x). All participants performed a pre-test and post-test on the VR simulator (the four basic tasks + one additional basic task). Halfway through the training protocol, both groups performed a salpingectomy on the VR simulator as interim test.

**Results:**

Both groups improved their performance significantly (Wilcoxon signed-rank, *P* < 0.05). The performances of group S and group M in the additional basic task and the salpingectomy did not differ significantly (Mann–Whitney *U* test, *P* > 0.05). Group S performed the four basic tasks in the post-test on the VR faster than group M (*P* ≤ 0.05), which can be explained by the fact that they were much more familiar with these tasks.

**Conclusions:**

Training of basic laparoscopic tasks on single or multiple modalities does not result in different training outcome. Both training methods seem appropriate for the attainment of basic laparoscopic skills in future curricula.

Laparoscopic surgery requires additional psychomotor skills on top of the skills needed in open surgery. These additional skills among others consist of handling longer instruments, counterintuitive movements of the instruments, and indirect view of the operating field. Laparoscopic basic skills are pre-eminently suitable to train in a preclinical setting. Previous research proves that training in a preclinical setting improves performance in the operating room [1, 2]. By acquiring basic skills in a preclinical setting, residents can concentrate in the operating room better at the performance of the actual procedure.

During past decade, there has been a lot of research about the preclinical training model. Different simulators have become available on the market and were validated to facilitate basic laparoscopic skills training [3–9]. However, the optimal implementation of simulators in training programs remains topic of discussion and investigation. Due to the implementation of European legislation (European Work Time Directive), which reduced trainee working hours [10], and the increased workload due to rising use of healthcare facilities, training time needs to be used as efficiently as possible. Therefore, it is important to make optimal and evidence-based use of the available simulators to ensure the highest possible training outcome.

Training centres often use a mix of different simulation tools in their courses, such as combining virtual reality (VR) simulators and box trainers. In general, we can make a distinction between multimodality training (training on different types of simulators) and single-modality training (training on one type of simulator). The literature suggests that box trainers and VR trainers are equally suitable to train basic laparoscopic skills [11]. Both options have their own advantages and disadvantages.

Single-modality training, for instance, on a VR simulator can be convenient for the trainer, because trainee performance can be easily tracked. In addition, VR training is suitable for independent training, because the simulator provides feedback through numerical scores at the end of every exercise. Previous studies show that a whole training curriculum can be based on one VR simulator [12]. Box trainers are in general less costly and are assumed to have better haptics. The literature suggests that trainees would choose a box trainer over a VR simulator if only one trainer was allowed [13].

Nevertheless, one of the problems in training of basic tasks on one simulator is to keep the trainee sufficiently engaged to practice the tasks deliberately. A mix of training modalities can possibly make the training more appealing for the trainees and help to prevent trainees’ boredom. Besides the possible prevention of boredom, the literature suggests that practice variability can improve training outcome and therefore gives another argument to diversify training by using a mix of training tools [14]. However, it is important to know that training outcome is not negatively affected by variation in training tools.

The purpose of this study was to compare single-modality training with multimodality training for acquiring basic laparoscopic skills. Is the training outcome affected by use of a mix of different simulation tools over the use of one single simulation tool?

## Materials and methods

### Protocol

**Fig. 1:**
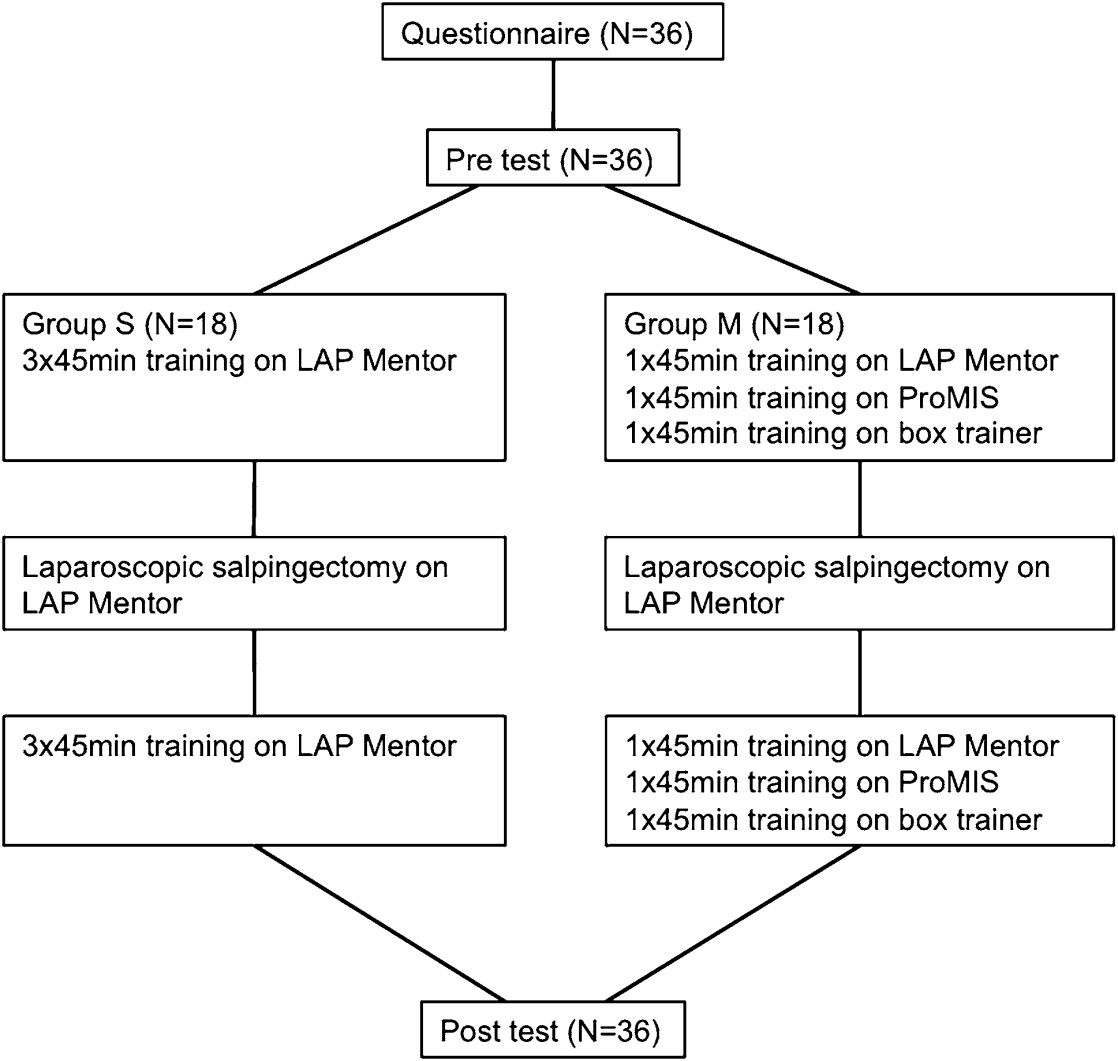
The training protocol; description of the tasks are provided in Table 1

In this study, 36 medical interns completed a training program of six sessions within 2 weeks (Fig. 1). In the introduction to the study, it was explained to the participants that the researchers were not affiliated with the manufacturer of the simulator and that all data would be analyzed anonymously. Informed consent was given by all participants (*n* = 36), after which they commenced the study by completing a questionnaire about demographics and prior laparoscopic or laparoscopic simulation experience. The participants were randomly divided in two groups. In total, all participants trained six times for 45 minutes. Group S trained single modality only on the LAP Mentor and group M trained multimodality on the LAP Mentor, the ProMIS III augmented reality simulator, and on a box trainer. The pre- and post-tests were the same for both groups (Table 1) and consisted of five different basic skills on the LAP Mentor in a set order. After the pre-test, group L continued training on the LAP Mentor on tasks 5, 6, 7, and 8 (Table 1). Group M continued training on three modalities: the LAP Mentor, the ProMIS III augmented reality simulator, and a box trainer. As an interim assessment and to keep the trainees motivated, both groups performed two full procedure salpingectomies on the VR simulator halfway through the training protocol after a video demonstration of the task. The first repetition was used to get familiar with the procedure; the second repetition was used for performance assessment. Tasks, such as the full procedure salpingectomy, are proven to be a valid tool to assess laparoscopic performance [12, 15].

**Table 1:** Performed basic tasks on the different simulators

			Pre- and post-test		Training	
Simulator	Task	Description	Group S	Group M	Group S	Group M
LAP Mentor	Task 5 Clipping and grasping	Grasp a leaking duct, stretch it until the red segment turns green, and place a clip on the green segment	✓	✓	✓	✓
	Task 6 Two-handed maneuvers	Locate the jelly mass, move part of the jelly aside until a red ball turns green, and pick up the green ball and put it in the basket	✓	✓	✓	✓
	Task 7 Cutting	Retract the form and cut the fibers in a circle	✓	✓	✓	✓
	Task 8 Electrocautery	Cut a highlighted band with a hook electrode; do not touch other bands	✓	✓	✓	✓
	Task 9 Translocation of objects	Lift the object and place it exactly into the transparent object matching the same color sides	✓	✓		
ProMIS	Instrument handling 1: Locating and coordinating	Touch and/or track a series of fixed and dynamic objects in a virtual environment				✓
	Instrument handling 2: Object positioning	Pick up a number of objects, transfer them from one hand to another, and place them in a specified target area				✓
	Instrument handling 3: Tissue manipulation	Stretch simulated tissue from one marked point to another				✓
	Dissection	Dissect a circle out of a stretched rubber glove				✓
Box trainer	Pipe cleaner	Place a pipe cleaner through 4 small rings				✓
	Placing rubber band	Stretch a rubber band around 16 nails on a wooden board				✓
	Placing beads	Place 13 beads to form the letter ‘B’				✓
	Cutting circle	Cut a circle from a rubber glove stretched around a plastic cup				✓

All basic tasks performed for pre- and post-test and the 6 × 45 min training sessions by the single modality (group S) and the multimodality group (group M)

### Equipment

The LAP Mentor II (Simbionix Corp., Cleveland, OH) is a VR-based laparoscopic training system. The software of the LAP Mentor II offers a variety of basic and procedural tasks in a VR environment to train different laparoscopy skills. After the performance of each task, the software provides numerical scores. In this study, we used five different basic tasks, and a full procedure salpingectomy case.

The ProMIS 3 is an augmented reality simulator (Haptica, Dublin, Ireland) for laparoscopic training. The laparoscopic interface consists of a torso-shaped mannequin. For each task, different trays must be placed in the mannequin. In this study, we used trays for four basic tasks (Table 1). The tasks were performed with AutoSuture disposable 5-mm Endo Clinch and Endo Shears (Covidien, Dublin, Ireland).

The box trainer that was used in this study was a trainer with an inanimate set, designed and developed by Leiden University Medical Centre (LUMC), The Netherlands [3, 16]. The simulator consists of a box with a non-transparent cover. It is equipped with a 30° scope (Karl Storz, Germany). The tasks were performed with Karl Storz laparoscopic instruments (Karl Storz). Four different basic tasks were used (Table 1).

### Statistics

All data were processed and analyzed using Statistical Package for the Social Sciences 18.0 for Mac (SPSS Inc., Chicago, IL). To analyse the differences in performance, the Mann–Whitney *U* test (between groups comparison) and Wilcoxon signed-rank test (within groups comparison) were used. A *P*-value < 0.05 was considered statistically significant. Due to technical problems, some participants of both groups (*n* = 9) trained task 9, the task preserved for pre- and post-test, during training sessions. In the analysis of the pre- and post-test performance, we separated these participants but included them in the analysis of the salpingectomy.

## Results

**Fig. 2:**
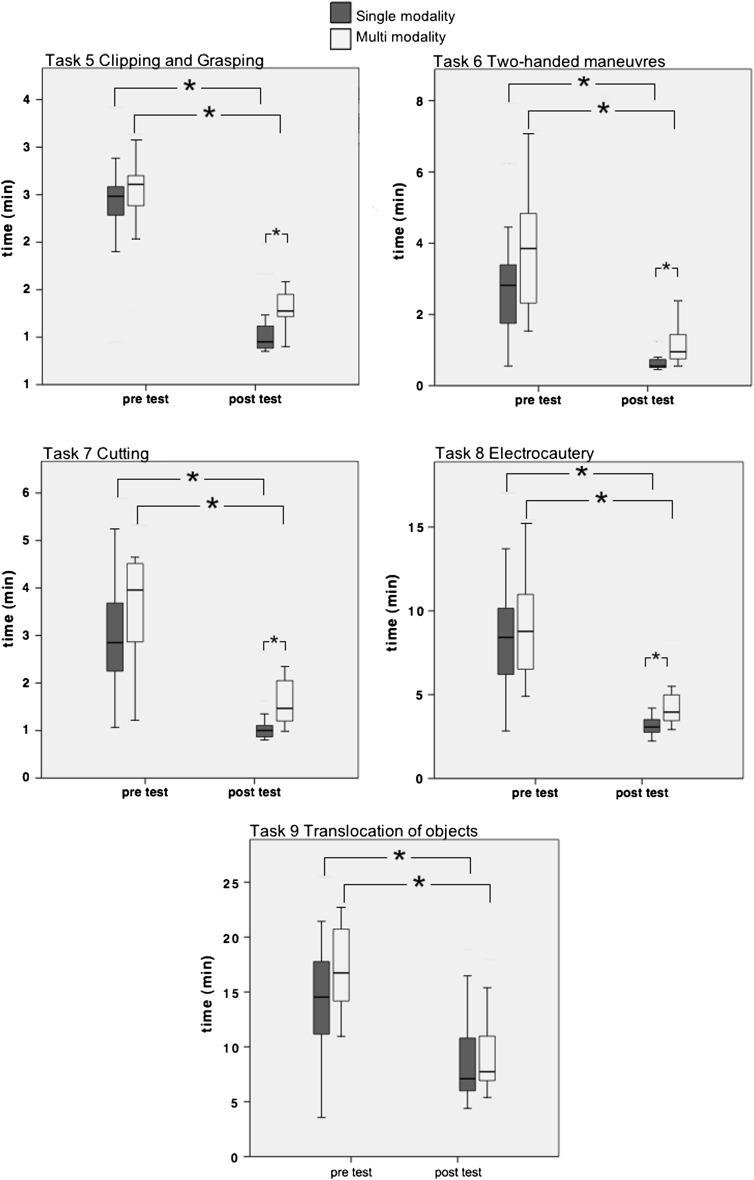
*Box* plots of time performance of the single modality group (*n* = 14) and the multimodality (*n* = 13) for the five basic tasks on the LAP Mentor, on pre-test and post-test. Within group analyses performed with the Wilcoxon signed-rank test and between the two groups with the Mann–Whitney *U* test (only the significant differences are presented). ^*^Significant difference of *P* < 0.05

Results of the questionnaire about demographics and prior laparoscopic experience did not differ significantly between the two groups; none of the participants had previous laparoscopic experience or laparoscopic training. At the pre-test, there were no statistically significant differences between the two groups for the parameters time, path length, and number of movements of the five basic tasks on the LAP Mentor. The performance level of both groups significantly improved when comparing the pre-test and post-test performance for the time, path length, and number of movement (Wilcoxon signed-rank, *P* < 0.05). On post-testing, the score time of group S was significantly better than those of group M, as well as for path length and number of movements for all tasks (*P* < 0.05), except for task 9. Figure 2 presents the performances of both groups in the pre- and post-test for the time to complete the basic tasks. None of the parameters of tasks 9 differed significant between the groups (Table 2). None of all parameters of the procedural salpingectomy assessment task differed significantly between the two groups (Table 3).

**Table 2:** Parameters of both groups (*n* = 27) on task 9, preserved pre- and post-test task and comparison of the groups in post test (Mann-Whitney *U* test)

	Single modality	Multimodality	*P* value
	Mean (min–max) (*n* = 15)	Mean (min–max) (*n* = 14)	
Time (min)	9 (4–19)	9 (5–18)	NS
No. of objects	6 (6–6)	6 (6–6)	NS
No. of properly placed objects	5 (5–5)	5 (5–5)	NS
No. of translocations	41.8 (21–105)	44 (13–92)	NS
Average no. of translocations per object	8.4 (4.2–21)	8.8 (2.6–18.4)	NS
Efficiency of translocations (%)	67.7 (22.9–100)	61.8 (26.1–100)	NS
No. of dropped objects	27 (10–71)	27.4 (7–78)	NS
No. of movements of right instrument	738.4 (366–1,210)	728.6 (422–1,630)	ns
No. of movements of left instrument	679.5 (290–1,344)	659.9 (390–1,156)	NS
Total path length of right instrument (cm)	2241.7 (1,180.1–4,067)	2,045.9 (1,013.2–5,607)	NS
Total path length of left instrument (cm)	2040.6 (975.4–3,959)	1,943.9 (760–3,936.7)	NS
Average speed of right instrument movement (cm/s)	2.9 (2.2–4.3)	2.6 (1.8–3.4)	NS
Average speed of left instrument movement (cm/s)	2.8 (2.0–3.6)	2.7 (2.1–3.3)	NS

*NS* not significant

**Table 3:** Parameters of both groups (*n* = 36) on the full procedure salpingectomy on the LAP Mentor and comparison of the groups (Mann–Whitney *U* test)

	Single modality	Multimodality	*P* value
	Mean (min–max) (*n* = 18)	Mean (min–max) (*n* = 18)	
Total time procedure (s)	317.2 (119–590)	273.6 (122–504)	NS
Idle time of right instrument	52.3 (5–133)	42.2 (10–129)	NS
Idle time of left instrument	65.4 (22–177)	55.1 (7–131)	NS
No. of movements of right instrument	279.5 (80–553)	243.9 (71–662)	NS
No. of movements of left instrument	282.6 (141–765)	236.6 (53–467)	NS
Total path length of right instrument (cm)	411.1 (101.9–867.5)	360.2 (104.8–849)	NS
Total path length of left instrument (cm)	395.6 (151.5–988.2)	347.1 (68.9–675)	NS
Average speed of right instrument movement (cm/s)	3 (1.9–5.7)	3.1 (2.2–4.5)	NS
Average speed of left instrument movement (cm/s)	2.8 (2.3–3.7)	2.9 (1.7–4.4)	NS
Minor bleeding incidents	7.5 (0–17)	6.9 (1–17)	NS
Injury to a vital structure	1 (0–7)	1.1 (0–6)	NS
Time electrosurgery is applied to treated fallopian tube (s)	3.3 (1.0–8.1)	3.2 (1.2–9.3)	NS
Ectopic pregnancy removed (%)	94 (0–100)	96.2 (0–100)	NS
Removal of resected specimen from the abdomen (%)	89.7 (0–100)	99.9 (99–100)	NS
Grasping anatomical structures with graspers or bipolar forceps	25.3 (9–62)	20.3 (8–45)	NS

*NS* not significant

## Discussion

The purpose of this study was to compare single-modality training with multimodality training for acquiring basic laparoscopic skills. In this study, we show that performance outcomes of training basic skills do not differ between multi- and single-modality training. Training results for both training methods did not differ significantly on the task that was preserved for pre- and post-test only. We confirm that novices can extensively improve their skills in basic laparoscopy by training on the LAP Mentor solely, but also on different training modalities, such as the ProMIS simulator and a box trainer. These results are in line with previous studies on basic skills training [17–22].

The fact that the single-modality group had better post-test performance scores than the multimodality group in tasks that both groups trained on can simply be explained by the significant difference in repetitions and training time on these specific tasks. The single-modality group trained a total of 6 × 45 min on these specific (LAP Mentor) tasks, and the multimodality group only 2 × 45 min.

Research in other fields than healthcare indicates that practice variability leads to better practice outcome [14]. This implies that we expected the multimodality group to outperform the single-modality group in the assessment tasks because of the higher level of practice variability. These results were not found in our study. Possibly, the specific simulator experience advantage on the VR simulator of the single-modality group levelled out the practice variability advantage of the multimodality group in the assessment tasks.

Previous research states that a VR simulator, such as the LAP Mentor, has the capability to incorporate the full laparoscopic training curriculum [12]. Our study can confirm that training on a VR simulator solely provides equal performance levels as multimodality training, and so training of laparoscopic basic skills can be performed on a VR simulator only.

On the other hand, this study shows that the training outcome of multimodality training is not inferior to the training outcome of single-modality training of basic laparoscopic skills. This implies that basic-skills training do not necessarily have to take place on one simulator. The choice of single modality or multimodality training can be led by other arguments, such as costs, convenience for the trainer, or opinion of the trainees [13].

Besides the results of the equal assessment performance level after training for both groups, our study shows another important result. Training tasks should only be used for assessment if exposure to the assessment task is equal among the tested subjects. The single-modality group eventually outperformed the multimodality group in the training tasks. The lack of simulator experience on these specific tasks was not compensated by the increased level of practice variability by the multimodality group. This suggests that the basic tasks on the simulator not only train and assess basic laparoscopic skills but also train simulator-specific skills that cannot be compensated by training on different simulation modalities. This is something to pay attention to when assessing skills at the end or beginning of a training program. Assessment tasks have to be equally available to the participants or specific unique assessment tasks have to be selected to assess objectively laparoscopic basic skills instead of assessing simulator experience.

Although this research provides the answer to the question of whether training outcome is affected by the use of a mix of different simulation tools over the use of one single simulation tool, more research is needed to compare the relative benefits and disadvantages of different simulation modalities and to extrapolate these findings to performance in the operating room.

In this study, we did not examine the influence of proficiency or criterion-based training. We recently addressed the importance of criterion-based training in a different study [23]. In the current study, criterion-based training would have influenced both groups equally and therefore would not have influenced the conclusion of equivalence of performance.

## Conclusions

Single-modality and multimodality training appears to have equal training performance outcome. In line of previous studies about laparoscopic surgical simulators, this study proves that training on simulators improves laparoscopic basic skills. The choice of single or multimodality training can be led by the available options. Both training methods seem appropriate for the attainment of basic laparoscopic skills in future curricula.

This research did not focus on retention of skills and transferability to the operating room. More research is needed to investigate retention and transferability. A longitudinal study is recommended to research long-term retention and transferability to the operating room.
